# Belonging to a Community—Newly Graduated Nurses' Lived Experiences of Participating in an Introduction Program: A Phenomenological Hermeneutical Study

**DOI:** 10.1111/scs.70238

**Published:** 2026-03-30

**Authors:** Anna Kjellsdotter, Fredrika Sundberg, Joanne Wills, Mia Berglund

**Affiliations:** ^1^ Research and Development Centre Skaraborg Hospital Skövde, Region Västra Götaland Sweden; ^2^ School of Health Sciences Skövde University Skövde Sweden

**Keywords:** lived experience, new graduate nurse, nursing, peers, phenomenological hermeneutic design, transition shock, transition to practice

## Abstract

**Aim:**

This study aimed to explore newly graduated nurses' lived experiences of transition while participating in a Clinical Nursing Introduction Program and to describe factors that influence personal and professional development.

**Methodological Design:**

A phenomenological hermeneutic design was used for researching lived experiences.

**Research Methods:**

The data comprised nurses' individual and group interviews (*n* = 14) and written narratives (*n* = 21) after completing the Clinical Nursing Introduction Program approximately 1 year after graduation. The data was analysed using phenomenological hermeneutical approach.

**Results:**

The lived experiences of transitioning while participating in the Clinical Nursing Introduction Program mean personal and professional growth, which, after 1 year, manifests as increased security and self‐confidence. The findings are presented in five themes: Belonging to a community, Stepping out of the comfort zone, The clinical wards' environment and atmosphere, Developing an allowing attitude, and Gaining confidence in profession and self.

**Conclusions:**

This study illuminates the pivotal role of the Clinical Nursing Introduction Program, comprising a community with peers, designated education days away from the ward, and group reflection sessions that facilitate the transition of newly graduated nurses into professional practice. These factors not only mitigate transition shock but also promote the development of competence and confidence, which are essential for personal and professional growth. The results also emphasised the need for ongoing group‐based reflective supervision, which is essential for promoting lifelong learning within the nursing profession. These findings from a hospital setting can be implemented for newly graduated nurses in other healthcare settings, such as Primary Care and Community care. By facilitating such supportive environments, healthcare organisations can ensure smoother transitions and empower newly graduated nurses (NGNs) to thrive in their roles, ultimately enhancing the quality of patient care.

## 1 Introduction

Over recent decades, nursing education has shifted toward a more theoretical and academic focus, sparking ongoing debates about the clinical skills required by graduates [1]. This shift has highlighted a gap between the theoretical foundations of nursing education and the practical competencies needed in practice [2]. Newly graduated nurses (NGNs) often feel uncertain about their professional roles and unprepared for the responsibilities of clinical practice, which include both clinical and supervisory duties [2, 3, 4]. To address these issues, nursing introduction programs have been implemented in hospitals in several countries [5].

## 2 Background

The World Health Organization [6] identifies the shortage of nurses as a critical healthcare issue. However, research shows that NGNs face numerous challenges transitioning from higher education to clinical practice [7, 8]. In nursing research, transition is defined as the process of moving from one life phase, condition, or status to another. It is often viewed as a period between stable states and a process that unfolds over time, divided into stages or phases [9, 10, 11]. The first year post‐graduation is crucial, as experiences during this time significantly impact NGNs' decisions to remain in or leave the profession [5, 12, 13]. During this year, NGNs may experience growth, development, and support from colleagues [14]. However, conflicts between personal principles and workplace values can lead to intellectual and emotional discord [15].

Kramer [16] described the conflict experienced by NGNs when expectations and reality differ as ‘reality shock’. Building on this, Duchscher [2] introduced the theory of ‘transition shock’ to illustrate the extensive changes NGNs face during their transition to professional practice. Research has identified several negative emotions associated with this transition, including attrition [17], isolation, feeling overburdened [7, 14, 18], and a lack of support [15]. These challenges contribute to a significant dropout rate among NGNs [15, 19]. To mitigate these issues and prevent dropout, healthcare organisations often implement introduction programs for NGNs after graduation [7, 8, 12, 20]. Despite extensive research, evaluating the effectiveness of specific approaches to nurse transition and retention remains challenging [12]. However, a supportive environment, including team integration and feedback opportunities, was often considered beneficial [12]. NGNs entering the profession anticipate vulnerability and fear of mistakes but also hope for belonging and support [21]. Their ability to cope during the initial clinical practice period depended on how supportive and accommodating the workplace was [15], and whether they were encouraged to ask for assistance when needed [22]. NGNs needed understanding regarding their insecurities and efforts to become independent [23]. Recognising NGNs as novice practitioners and supporting their learning was crucial [24]. There is a consensus on the need for supportive environments to ease the transition and promote NGNs' growth and development [25], although few studies have focused on their experiences and perceptions regarding the transition to professional practice [5].

Literature reviews on introduction programs for NGNs often focus on a limited number of countries and examine their impact on patient care skills, retention, or turnover. There is a lack of conclusive evidence suggesting that these programs positively affect the transition experience [5]. A review of qualitative studies on transition programs found that while clinical readiness of NGNs was ensured, non‐clinical needs such as socialisation, professional growth, and support were often unmet [26]. The review highlighted the need for future research to explore NGNs' experiences in other countries, as the existing studies were primarily from Australia and the United States.

Research indicates that NGNs consistently encounter challenges during their transition into the nursing profession, underscoring the need for more qualitative research to understand this transitional process [5, 14]. Therefore, this study aimed to explore NGNs' lived experiences of transition while participating in a Clinical Nursing Introduction Program and to describe factors that influence personal and professional development.

## 3 Methods

### 3.1 Design

A phenomenological hermeneutical approach for researching lived experience, as described by Lindseth and Norberg [27], was applied. This approach was chosen to describe and interpret lived experiences of the newly graduated nurses. The study adhered to the guidelines of the consolidated criteria for reporting qualitative research (COREQ) checklist (Data S1) [28].

### 3.2 The Clinical Nursing Introduction Program

The Clinical Nursing Introduction Program (CNIP) was introduced in 2016 at a general hospital in southwest Sweden [20, 21]. Its purpose was to create a safe and supportive environment for NGNs to transition into the profession through education, clinical supervision, and critical reflection. The CNIP comprised five core components: employment and organisation, an introduction week, placements in different clinical settings, education days, and process‐oriented nursing supervision (POH), as presented in Table 1 [20]. The program had two admissions per year and ran for 14 months, with 30 to 45 NGNs participating in each admission.

**TABLE 1:** Core components of the Clinical Nursing Introduction Program (CNIP).

Employment and organisation	Newly graduated nurses (NGN) are permanently employed and work independently in the CNIP. After completing the CNIP, the NGN is offered employment at a care unit based on the NGN's choice and the unit's needs. The CNIP organisation, unit manager, program manager and administrator make continual changes and improvements to the program based on evaluations
Introduction week	NGNs receive lectures and information about the CNIP, employment at the hospital and the overall organisation. NGNs receive practical training, are introduced to administrative systems and collect necessary authorisations. Moreover, NGNs take part in cardiopulmonary resuscitation training and participate in a seminar on group dynamics
Placements in different clinical settings	NGNs' preferences, combined with the hospital's needs, determine suitable wards for the clinical rotations. On each ward, the NGN has an introduction and is assigned a senior registered nurse (RN) as a supervisor for at least 4 weeks. Furthermore, an experienced RN monitors the NGN's clinical progress in an ongoing process similar to mentorship. There is even the possibility of a one‐week observation period on another ward during each placement. Two placements, lasting 6 to 8 months, provide professional competence. The longer placement runs through the summer period
Education days	During the CNIP, NGNs enjoy a range of challenging environments and educational innovations that incorporate suitable levels of theoretical knowledge and practical skills to support them for practice readiness. NGNs also partake in 15 days of theoretical and practical education on current topics and receive training about working with a person‐centred approach
Process‐oriented nursing supervision (POH)	POH is a method whereby personal experiences from situations in healthcare are thought about and examined in a structured process where the ethical patient perspective and nursing values constitute the framework for reflection. During the CNIP, NGNs meet in their POH group on eight occasions and work with patient‐ and work‐related situations. Each POH group, with its own supervisor and six to eight participants, constructs an agreement outlining how to uphold confidentiality and contribute to an atmosphere characterised by openness, responsiveness, curiosity and acceptance

### 3.3 Participants and Setting

After completing the CNIP at a general hospital in Southwestern Sweden, all nurses were invited to participate in the study. A total of 35 nurses, aged 23 to 54 years, participated. Of those who completed the CNIP in 2018, 21 provided written narratives (18 females, 3 males). Nurses who completed the program between 2019 and 2020 were invited to participate through group or individual interviews, with 14 agreeing to take part. This resulted in three group interviews with 10 female participants and four individual interviews with four female nurses. All participants received written and verbal information about the research project and were informed that participation was voluntary, with no negative consequences for withdrawing at any time.

### 3.4 Data Collection

Data were collected after participants completed the CNIP through a combination of individual and group interviews, as well as written narratives. Group interviews were conducted in person at a conference center near the hospital, while individual interviews were conducted by telephone or via Zoom. The interviews were performed by the authors M.B., J.W., and research nurses at the local hospital.

The interviews were conducted in a dialogue format, starting with the question, ‘Please describe how it feels for you now to be a nurse with one year's work experience?’ Follow‐up questions were added when appropriate, for example, ‘How have you developed during this time?’, ‘What has been particularly challenging or supportive?’, and ‘Can you describe your experiences of the CNIP?’ Open‐ended questions and probes were used to encourage participants to reflect on and discuss their experiences. Follow‐up questions like ‘What do you mean by that?’ allowed participants to provide more detailed responses, offering a broader, more complete perspective on their experiences and enabling interviewers to verify their understanding of the phenomenon. The interviews, lasting between 34 and 63 min, were recorded and transcribed verbatim.

The written narratives, where nurses described their experiences, were based on following questions: ‘How do you view your professional and personal development during the year in the CNIP?’ and ‘What has affected your development?’. Each narrative was approximately one A4 page of handwritten text, and the responses to each question were grouped together.

### 3.5 Data Analysis

The phenomenological‐hermeneutical approach, as described by Lindseth and Norberg [27], inspired by Ricoeur's theory of interpretation [29] and enables a deeper understanding of the phenomenon in focus. Ricoeur describes interpretation as a hermeneutical movement between the whole and the part and between understanding and interpreting the text.

The analysis process conducted by all authors comprised three phases: *naïve reading, thematic structural analysis* and *comprehensive understanding*. *Naïve reading* involved several readings to grasp the overall meaning of the text. *Thematic structural analysis*: first, the text was read several times to gain an overall understanding of its content. Next, the text was divided into meaning units that expressed essential aspects of the participants' experiences. These units were then condensed to preserve the core meaning in a more concise form. In the following step, the condensed units were abstracted, which involved identifying underlying meanings beyond the literal expressions. The abstract units were then compared based on similarities and differences, enabling systematic grouping. Through this process, themes were formulated to represent the fundamental structures of the participants' lived experiences. Finally, the analysis resulted in five overarching themes that summarise the existential meaning of the studied phenomenon. Themes were reviewed to validate the initial understanding, with participant quotations (both from interviews and narratives) included to validate interpretations (Table 2). The final phase, *comprehensive understanding*, summarised and reflected on the themes in relation to the research question and study context. It involved critical reflection to achieve a deeper understanding of the phenomenon, considering the naïve understanding and validated themes alongside the researchers' preunderstanding as nurses. The authors, who are lecturers in nursing education, are involved in the CNIP as reflective supervisors. The most prominent theories used in this step of the analysis were the life‐world theory of learning, support for learning (life‐world didactic) [30, 31] and Duchscher's theory of transition [2].

**TABLE 2:** Examples of the thematic structural analysis of the meaning of transition into nursing while participating in a CNIP.

Meaning unit	Condensation	Theme
In the CNIP, I met other NGN. We reflect together; everyone has similar experiences. It's nice to feel that you're not alone	Safety in the recognition factor	Belonging to a community
Changing placement is tough and an enormous struggle for me, but to be able to look back on what I went through and learned from this has been positive and an incredible development	Challenge revealed as transformative	Stepping out of the comfort zone
Of course, all my colleagues have been supportive when I dared to ask, but the sense of security has always been there—I am not alone in this	Colleagues' support gave safety and belonging	The clinical wards' environment and atmosphere
Awareness that you know enough, but with a humility that you do not know everything and still think this is ok	Awareness of one's attitude toward oneself	Developing an allowing attitude
I feel safer and more secure in my professional role. Grown both professionally and personally. Proud of where I've come	Safer and more secure in the professional role	Gaining confidence in profession and self

### 3.6 Ethical Considerations

The study received approval from The Swedish Ethical Review Authority (No. 1056‐18) and adhered to the principles of the Declaration of Helsinki [32]. Written informed consent was obtained from all participants, with permission to use quotations in the results. Participants were informed that they could withdraw from the research project at any time without providing a reason.

## 4 Findings

### 4.1 Naïve Understanding

The employment and organisation within the CNIP, which includes education days, POH, and two different clinical placements, create a safe and secure framework for transition. The lived experiences of CNIP mean personal and professional growth, which, after 1 year, manifests as increased security and self‐confidence. This involves a deeper understanding of various aspects of nursing and how NGNs can influence both their work roles and patient care. However, the transition is also challenged by the program's requirement of two different clinical placements, which necessitates starting over again. The climate of acceptance for being new in a community with other NGNs that CNIP provides is a meaningful and crucial factor, along with being challenged and supported. Participating in the CNIP increases self‐confidence, changes initial high demands on oneself and perceptions of the nursing role, self‐insight occurs, and the need and desire for continuous development transpire.

### 4.2 Thematic Structural Analysis

The thematic structural analysis resulted in five themes (Table 3).

**TABLE 3:** Themes from the structural analysis.

Transition while participating in a CNIP
Belonging to a community
Stepping out of the comfort zone
The clinical wards' environment and atmosphere
Developing an allowing attitude
Gaining confidence in profession and self

### 4.3 Belonging to a Community

This sense of belonging to a community was experienced in various forums within the CNIP. Through POH and within that group, NGNs met peers and experienced a shared recognition of being new together, offering opportunities for joint reflection and education. The specific employment in CNIP that incorporated POH provided legitimate occasions to get away from the ward and join an accepting and understanding community to share thoughts and reflect with others in the same situation: *It's been good to be able to discuss with this group situations that have been difficult, professionally and personally. It makes it easier to process situations*. Having time to reflect together and learn from each other without pressure to meet ward expectations supported NGNs' development. *A large part of my development is just the CNIP and POH, meeting other new nurses and getting through the first tough year together*. Belonging to the POH community reassured NGNs that they were not alone in being new, and it strengthened their courage to trust each other: *It creates a greater sense of security to dare to talk openly about difficult things and learn from each other's experiences*. The opportunity to reflect with peers from different backgrounds also enhanced their ability to understand and explore alternative courses of action. *POH has been a free, accepting and understanding environment for reflection. It has also contributed to me now daring to stand up and ask questions*. The conversations also contributed to a deeper understanding of oneself and one's future professional role. Additionally, these reflective conversations helped strengthen the link between theoretical knowledge from university and practical experience: *Reflection—it's been a good way to connect school with working life—the common thread continues*.

### 4.4 Stepping Out of the Comfort Zone

Through the experience of two consecutive placements, intentionally ordered, NGNs encountered profound opportunities for growth by stepping out of their comfort zones. Changing placements immersed professional realities, as NGNs navigated the distinct challenges of varied wards and patient groups. Simultaneously, they grappled with the personal experiences of being new—confronting unfamiliar routines, adjusting to different workplace cultures, and forming new connections with colleagues. Stepping out of the comfort zone embodied a movement between the discomfort of starting over and the exhilaration of discovery. While these experiences were often challenging and time‐consuming, they became meaningful milestones in the NGNs' professional and personal development, shaping their identities and confidence as practitioners. *It's rewarding to change wards, even if it has been tough and demanding at times*. Initially uncertain about what to expect, NGNs gradually adapted to their new environment. Initially uncertain about what to expect, NGNs gradually adapted to their new environment. Over time, they gained self‐confidence and developed an increasing ability to articulate their opinions and thoughts: *Having to start on new wards and try to fit in and get to know them is strenuous. Now, I feel comfortable with most people around me, which makes it easier to think and dare to say what I feel. It's also helped me out of work*.

Through the experience of changing placements, awareness of personal and professional growth emerged, along with valuable insights into the knowledge and skills acquired: *Changing placement is tough and an enormous struggle for me, but to be able to look back on what I went through and learned from this has been positive and an incredible development*. Entering the second placement, NGNs found themselves starting anew to some extent but with the advantage of accumulated knowledge and skills from their first placement. This transition provided opportunities to deepen their understanding through exposure to diverse situations and assignments across both placements. These experiences encouraged personal reflection on actions and thoughts, facilitating learning by intertwining new insights with existing knowledge. As a result, their confidence grew considerably.

### 4.5 The Clinical Wards' Environment and Atmosphere

The specialties and specific atmospheres of clinical wards, shaped by various factors, played a pivotal role in the transition and learning opportunities for NGNs. These specialised areas influenced the transition process during the first year. In placements within specialist wards like cardiology, NGNs experienced a sense of in‐depth learning and described it as having ‘*become good at one thing*’. Moreover, placements across two wards within the same specialisation facilitated smoother transitions. Conversely, placements in different areas presented challenges to the overall transition.

Learning was both facilitated and hindered by the ward's organisation. Access to a supervisor and supportive colleagues, who provided feedback, affirmation, and time for reflection, created a ‘safe harbour’ and offered the opportunity to consolidate learning: *It was nice to have someone who could give feedback, who could listen to my thoughts*.

A supportive collegial atmosphere promoted a sense of ease, where stress was alleviated, questions were encouraged, and there was less pressure on themselves. The presence of peers, when several NGNs worked together in the same ward, brought a sense of shared understanding and mutual support that further enriched the experience. In contrast, an inadequate introduction, ongoing organisational changes, unclear leadership, and the absence of sufficient mentorship created an unsettling environment that hindered NGNs' development. Insufficient recovery weakened NGNs' ability to shoulder work responsibilities and carry out their tasks, leading to a feeling of unsafety.

The full‐day structure education days within the CNIP created a sense of freedom from ward responsibilities, offering an environment that promoted focused learning and reflection: *Good with a full day, then you know that today I don't have to think about the ward, but I just focus on the lecture or reflection*.

External circumstances, such as an ongoing pandemic, shaped the ward's atmosphere and impacted NGNs' learning in various ways. A high workload demanded rapid and continuous acquisition of new knowledge, often creating poorer conditions for support: *The Covid situation consumed everyone—bad mood, less support, and new guidelines twice a day*. The unique circumstances of the Covid pandemic during NGNs' first year placed both experienced staff and novices in an unfamiliar situation: *Everyone was new to the situation with Covid. It welded us together and we gave support to each other*. This shared experience strengthened the staff and provided conditions for mutual support.

### 4.6 Developing an Allowing Attitude

NGNs experienced a profound transition during CNIP, both in their professional roles and self‐perception. Initially overwhelmed by the high pace, administrative burdens, and immense responsibilities of nursing, they realised the impossibility of being omniscient or faultless. This shift means moving away from perfectionism toward self‐compassion. Through CNIP education days, nursing as continuous learning became visible when adopting a more accepting attitude toward personal knowledge. Embracing an attitude that acknowledges their competence as sufficient for safe practice while remaining humble about what they had yet to learn. ‘*Awareness that you know enough, but with a humility that you do not know everything and still think this is ok*’.

When experienced colleagues acknowledged learning from novices, NGNs began to recognise that learning is mutual and that professional growth continues for everyone, regardless of experience. This realisation eased their self‐imposed demands as their confidence in their abilities grew. They began to trust themselves more and adjusted their understanding of what constituted a reasonable work effort. With improved planning, they were able to reprioritise tasks, creating space to focus more on building patient relationships.

NGNs learned to separate work from their private lives: ‘*I've learned to let go of work when I go home. I can feel calm that I've done my best during my shift*’.

NGNs set boundaries, realising the limits of the workday and that responsibility could be shared. Through delegation and collaboration, they reduced self‐imposed demands, focusing more on their health and finding balance between work and leisure. ‘*If I do something after work—train, meet a friend, it's easier to let go of the job—then you usually stop feeling tired’.*

### 4.7 Gaining Confidence in Profession and Self

Each step NGNs took during their placements in CNIP, including POH and educational days, contributed to their personal and professional growth. Learning progressed through time spent on the wards, which led to a deepened sense of confidence in their profession. Experience from the CNIP provided a foundation for NGNs, leading to knowledge about their strengths and limitations that facilitated their confidence. These insights contributed to feelings of security and acceptance of their need for support and the courage to ask for help. The first year as a nurse meant going through numerous stages of growth, and a feeling of relief was experienced when it was over. Initially, a lack of belonging was felt and trust in their knowledge, as new situations were challenging. However, as time progressed, insights into the evolution of their personal and professional roles were gained. ‘*You become more and more confident in your role. You undergo a great deal of development during the year, both professionally and on a more personal level*’.

After a year, the novelty wore off, and increased self‐confidence led to reduced sensitivity to criticism. The feeling of being both new and experienced emerged. When other graduates entered the profession, nurses with 1 year of work experience were, in comparison, considered capable, even though NGNs realised that they still had a lot to learn. Interacting with less experienced colleagues highlighted how much they had grown and learned.

Confidence in the nursing role grew through varied ward experiences, supportive colleagues, collaboration in interprofessional teams and patient interactions. This progression was marked by a sharper clinical eye, greater sensitivity with patients, and an improved ability to apply knowledge. Increased confidence was seen in taking on professional responsibility and making decisions with greater ease. ‘*Now, I feel confident in my role and trust more in me and my knowledge. I have learned to handle different situations’.*

NGNs became more confident in offering opinions during rounds, practicing technological procedures, and handling medication. Their growing ability to listen to and interact with patients boosted their confidence, leading to feelings of pride and happiness both at work and in their personal lives. ‘*I've developed and am more independent, more secure, and very satisfied. I like myself and the job more*’.

## 5 Comprehensive Understanding and Discussion

The naïve understanding and thematic structural analysis are explored in this comprehensive understanding through reflections grounded in (1) the life‐world perspective emphasises the importance of lived experience, highlighting that learning is not just about acquiring knowledge but about integrating it into the self and the world. Reflection on experiences is central to life‐world philosophy of learning [30, 31]. (2) Duchscher's theory [2], on the other hand, provides insight into the phases of transition, from novice to advanced beginner, and stresses the importance of reflection and mentorship in supporting this growth.

From a life‐world perspective, the experiences of transition via CNIP become apparent through the awareness that knowledge, such as practical experience and a deeper understanding of caring and personal development, has been incorporated into them as persons and as nurses. The interaction between learning and caring, theoretically, and practically, is deepened and intertwined with the support of the CNIP into a professional approach in the caring role but also in relation to the demands made on oneself. This learning includes the recognition that they now think and act as independent nurses, a learning process that results in more realistic pressures on and expectations of themselves.

NGNs' learning and gradual development toward increased independence can be supported by the structure that the CNIP provides and the community among nurses. After 6–8 months in a clinical ward, when NGNs have become secure in their role, the challenge of changing wards and thus, for a time, reverting to novicehood again also contributes to the learning process. The question is whether the CNIP accelerates and supports growth or adds stresses to NGNs? Being challenged to take the next developmental step can also be understood based on what Duchscher [2] describes as mentally moving into the next phase of learning that is, being an advanced beginner [33].

The NGNs in this study go through a progression from a novice to an advanced beginner [2]. The phases of transitions to practice are not linear, nevertheless they consists of *doing* (3–4 months), *being* (4–8 months) and *knowing* (9–12 months), where the ability to focus more overall develops at the end of the *being* phase [3]. Changing wards at this point could conceivably be a disturbing factor for NGNs' development and independence, but NGNs describe this as particularly significant for their development and learning to be a learning person. A factor that might be important is that nurses who partake in the CNIP have already been trained from their student days to take their starting point for care from the patient's perspective and to have a holistic view of the patient's situation.

These findings are in line with what Duchscher [2] described as transition shock when transitioning from student to clinical nurse. Introduction programmes for NGNs can reduce stress [26] and support the process of developing competence and confidence in clinical work and decision‐making [34]. This study also identified factors and components that are needed to get past these overwhelming emotions, such as support from colleagues and clinical experience. Participating in the CNIP and the sessions with critical reflection was a major part of this process, providing platforms for reflection, sharing experience, and learning from peers facing similar situations. The sense of community and the opportunity to share experiences contributed to the NGNs' ability and willingness to ask questions and seek support when needed. Previous research has similarly shown that introduction programmes are necessary for creating direction, support, and motivation, as well as coping with professional and psychological challenges that NGNs face in their daily work [35].

NGNs are confident in the caring science knowledge base and question care in a new way. This can be extra challenging but also something that contributes to developing care to become more person‐centred. The interaction between learning and caring is supported by group reflection based on patients' narratives [31]. Our findings align with several studies, showing that joint reflection, like POH, is a key factor that enables nurses to show courage and stand up for the theoretical basis, the profession, and patients [30, 36, 37].

Support for NGNs' learning comprises the allocated time and sense of community with other NGNs that training days in the CNIP and group supervision (POH) provide. The ability to step back from daily tasks is a successful factor and imperative for entering the described development process [31]. According to the results, reflection contributes to relief and concurrently gives the courage to stand up for the patient, oneself as a nurse and oneself as a person. Self‐confidence develops with willingness and understanding the importance of being in a constant state of learning, while simultaneously allowing oneself to rest. Development in the professional role thus affects the nurse as a private person, demonstrating the importance of including the whole person in the learning process, something that can be understood from a life‐world perspective.

The interviewed nurses emphasised the importance of experience and exposure to diverse situations in building confidence and competence in transitioning and becoming nurses. As they gained more experience with the clinical environment, their professional progression evolved, and they developed clinical skills and acquired the ability to manage more responsibilities and make informed decisions. Support from colleagues, collaborations with other professions, working together in teams, and interactions with patients also contributed to the transition and provided a sense of confidence and work satisfaction.

The findings also showed a shift in the NGNs' mindset, as they learned to accept that perfection is not the goal and to recognise that continuous learning is an essential part of the nursing profession. This change in attitude enabled the NGNs to be more confident, humble, and open to learning from individuals of all experience levels. The transition to becoming a confident nurse is a multifaceted process that encompasses personal growth and professional development through supportive environments.

## 6 Methodological Considerations

This study, using a phenomenological hermeneutical approach based on interviews and written narratives, acknowledges that the interviewers' understanding of the research topic may have influenced the questions and analysis [27]. To enhance credibility, the analysis process was conducted and validated collaboratively by all authors. To enhance confirmability, we reflected on and discussed the themes during the structural analysis to validate our naïve understanding and prevent confirmation bias, ensuring findings were grounded in the data [27, 38]. The interview sessions facilitated an open environment, allowing participants to share their experiences freely. The interviewers' extensive experience in clinical practice and qualitative research contributed to the richness of the data by creating an open and trusting dialogue, inviting participants to expand their narratives and share their lived experiences in depth. Phenomenological research requires a critical and reflective approach, balancing parts and the whole to ensure validity. In this process, we tried to bridle our understanding to ensure validity [27]. In the comprehensive understanding, the findings were reflected upon with our preunderstanding and chosen literature in mind. An open, critical discussion among all authors was employed to ensure validity. Participation in the CNIP is mandatory, but participation in the study was voluntary. The methodology of phenomenological hermeneutical does not seek a single truth but acknowledges multiple interpretations. There are always possible interpretations and various ways of arguing for and against them [29, 30]. As with all qualitative studies, the transferability of results to similar contexts and other countries must be assessed by the reader.

## 7 Conclusion

The findings in this study illuminated the significance of the CNIP. Supportive factors such as being in a community of peers with time for reflection, being challenged in changing clinical wards, and full education days were experienced. The presence of encouraging colleagues and a supportive work environment facilitated a smooth transition for newly graduated nurses as they embarked on their professional journey. The findings also revealed a need for ongoing reflective supervision in groups as essential for the nurses' ability for lifelong learning in their profession. The uniqueness of this CNIP was the employment and organisation that gave the nurses sanctuary from the daily demands and therefore eased the transition. We argue that CNIP can be implemented for newly graduated nurses in other healthcare settings, such as Primary Care and Community care. By facilitating such supportive environments, healthcare organisations can ensure smoother transitions and empower NGNs to thrive in their roles, ultimately enhancing the quality of patient care.

## Author Contributions

A.K. and M.B. were substantially involved in the study design and in the development of the research questions and focus group guides. A.K. directed the data collection, while M.B. and J.W. conducted the interviews together with the research nurses. All authors contributed to the data analysis, were involved in revising and confirming the themes, and participated in writing the manuscript. A.K., F.S., and M.B. prepared the manuscript for journal submission. All authors meet the criteria for authorship and have approved the final version of the article.

## Funding

This research did not receive any specific grant from funding agencies in the public, commercial, or not‐for‐profit sectors. The study was conducted with support from the Research and Development Centre, Skaraborg Hospital, the University of Skövde, and the Skaraborg Institute, Sweden.

## Conflicts of Interest

The authors declare no conflicts of interest.

## Data Availability

The data that support the findings of this study are available on request from the corresponding author. The data are not publicly available due to privacy or ethical restrictions.
